# Selecting Evidence-Based Content for Inclusion in Self-Management Apps for Pressure Injuries in Individuals With Spinal Cord Injury: Participatory Design Study

**DOI:** 10.2196/15818

**Published:** 2020-05-20

**Authors:** Maddalena Fiordelli, Claudia Zanini, Julia Amann, Anke Scheel-Sailer, Mirjam Brach, Gerold Stucki, Sara Rubinelli

**Affiliations:** 1 Institute of Communication and Health Faculty of Communication Science Università della Svizzera italiana Lugano Switzerland; 2 Swiss Paraplegic Research Nottwil Switzerland; 3 Department of Health Sciences and Medicine University of Lucerne Lucerne Switzerland; 4 Department of Health Sciences and Technology Health Ethics and Policy Lab ETH Zürich Zürich Switzerland; 5 Swiss Paraplegic Centre Nottwil Switzerland

**Keywords:** mHealth, paraplegia, tetraplegia, pressure ulcers, consensus meeting, community engagement, recommendations

## Abstract

**Background:**

Technological solutions, particularly mobile health (mHealth), have been shown to be potentially viable approaches for sustaining individuals’ self-management of chronic health conditions. Theory-based interventions are more successful, as evidence-based information is an essential prerequisite for appropriate self-management. However, several reviews have shown that many existing mobile apps fail to be either theoretically grounded or based on evidence. Although some authors have attempted to address these two issues by focusing on the design and development processes of apps, concrete efforts to systematically select evidence-based content are scant.

**Objective:**

The objective of this study was to present a procedure for the participatory identification of evidence-based content to ground the development of a self-management app.

**Methods:**

To illustrate the procedure, we focused on the prevention and management of pressure injuries (PIs) in individuals with spinal cord injury (SCI). The procedure involves the following three steps: (1) identification of existing evidence through review and synthesis of existing recommendations on the prevention and self-management of PIs in SCI; (2) a consensus meeting with experts from the field of SCI and individuals with SCI to select the recommendations that are relevant and applicable to community-dwelling individuals in their daily lives; and (3) consolidation of the results of the study.

**Results:**

In this case study, at the end of the three-step procedure, the content for an mHealth intervention was selected in the form of 98 recommendations.

**Conclusions:**

This study describes a procedure for the participatory identification and selection of disease-specific evidence and professional best practices to inform self-management interventions. This procedure might be especially useful in cases of complex chronic health conditions, as every recommendation in these cases needs to be evaluated and considered in light of all other self-management requirements. Hence, the agreement of experts and affected individuals is essential to ensure the selection of evidence-based content that is considered to be relevant and applicable.

## Introduction

### Background

Ever since communication technologies were adopted for health care purposes and defined under the umbrella term electronic health (eHealth), the concept of empowerment and the use of technological solutions have become intertwined [1]. As technological devices became more personal and connected, this relationship took on a new relevance. In particular, mobile health (mHealth) solutions, commonly defined as the use of mobile and wireless technologies to support the achievement of health objectives [2], have been used to enhance the self-management of various chronic conditions [3], such as diabetes [4] and asthma [5]. Evidence indicates that these mHealth solutions can foster self-management by addressing multiple risk factors [6] and sustaining long-term adherence to prevention measures, which remains a major issue [7].

Studies have examined not only the effectiveness of mHealth [8], but also its design and development process. Even though there is great potential for using mobile technologies for health purposes, findings show that many of the existing mobile apps are not theoretically grounded [9] and their contents are not based on evidence [3,7,9,10]. This is problematic because studies mentioned that theory-driven health interventions are more effective than those without theoretical grounding [11]. It is only recently that mHealth has started to adopt strategies informed by behavior change theories, but this adoption has not been systematic [12]. Some apps are only partially applying the principles of behavior change theories [13], whereas others have defined the app’s features or its mechanism (ie, goal setting) based on a set of different theories or models, but without clear reference to them [14-16]. Additionally, some authors have attempted to create a framework to develop digital behavior change interventions that integrate, for instance, behavior theories, design thinking, and user-centered design [17,18]. Despite these efforts, to date, many apps are still not based on theory, as attested by recent systematic reviews [19,20].

Another flaw of many existing apps lies in the quality of their content, which does not reflect the latest scientific evidence. Indeed, some ex-post examinations of mHealth apps underlined that their content rarely adheres to evidence-based knowledge [21-24]. Some authors have based the content of their interventions on the results of systematic reviews or additional participatory efforts (ie, involving different stakeholders) [25,26]. However, their procedures are not detailed and cannot be replicated. So far, the efforts to develop a framework for integrating evidence-based content into mHealth interventions have been limited [27]. Hence, it remains unclear how disease-specific recommendations and professional best practices should be selected to inform mHealth interventions. This is problematic as evidence-based information can enhance health literacy [28], which is a precondition for patient participation and informed decision-making [29]. Consequently, apps that are based on outdated or inaccurate content might negatively affect the users’ health and safety [30-32]. Considering the huge amount of incorrect and misleading information available on the internet, as well as in leaflets and other lay publications [33,34], it is of utmost importance that new mHealth interventions tackle the issue of content quality.

Participatory design is a democratic process involving different stakeholders from the early phases of the design process [35-37]. At least the following two premises provide the basis for different participatory design approaches: all stakeholders should be involved in the design phase to inform the approach and this will increase the likelihood of technology acceptance because it will help set clear expectations [38]. It is for a very good reason that many authors underscored the potential of a participatory design approach throughout various steps, such as requirement analysis, definition of features, and user interface design [39-41], but without providing much clarity on the most appropriate involvement of experts and other stakeholders for the selection of content. Participatory design could be a viable approach for achieving the evidence basis of an app. Several guidelines exist, but they are mostly designed for health care professionals rather than for community-dwelling individuals or patients. Selecting the content of an app through a participatory design approach involves understanding which of the recommendations are not only impactful in terms of prevention, but also feasible and applicable for people living in the community.

The objective of this study was to fill this gap by describing a structured procedure for the participatory identification of evidence-based content to ground the development of a self-management app. To illustrate the procedure, we used a project based in Switzerland aiming to develop an app for the prevention and self-management of pressure injuries (PIs) in individuals with spinal cord injury (SCI).

### A Case in Point: Spinal Cord Injury

SCI is a complex chronic condition affecting human functioning in all aspects [42], and it is associated with a number of complications [43,44]. People with SCI have a high risk of developing PIs [42]. The incidence of PIs in the SCI population is 25% to 66% [45], and approximately 85% of individuals with SCI will experience PIs at some point in their lifetimes [46]. PIs impact the quality of life of the affected individuals, as their treatment necessitates prolonged inactivity, which often results in a loss of income and a feeling of social isolation [47]. Moreover, evidence shows that PIs can account for approximately one-fourth of the cost of care for individuals with SCI [48].

There is general agreement on the fact that PIs might be often preventable in individuals with SCI [49] and that prevention is more cost-effective than treatment [50]. Prevention is possible through active self-management. However, this self-management remains challenging owing to the many different factors that need to be taken into account. Indeed, individuals with SCI have to play an active role in the prevention of PIs by, for instance, adapting their behavior, which includes repositioning, performing pressure-relieving movements, and keeping the skin clean [51]. The prevention and management of PIs in individuals with SCI could benefit from the development of an evidence-based mobile app that supports individuals in performing the many preventive measures, as well as monitoring and treating early stage PIs.

## Methods

### Study Design

We used a consensus method for the participatory identification of evidence-based content to ground the development of a self-management app for PIs in individuals with SCI. Indeed, to ensure that individuals with SCI have access to sources of information that are credible, of good quality, and up to date, the information provided in the app should be consistent with the latest available clinical recommendations, including those that are indeed the best available evidence for pressure ulcer prevention and remain the foundation of a prevention program [52].

The recommendations were identified through a three-stage research procedure developed following the main steps of the consensus development method [53]. First, a review of existing recommendations for the prevention and management of PIs in individuals with SCI was conducted. Second, a consensus meeting [54] to select the most important recommendations that individuals with SCI should apply in their daily lives was performed. Finally, the results were consolidated by the expert team. Both the review of the recommendations and the consensus meeting were conducted at the end of 2017, while the third phase was performed during the first quarter of 2018.

### Stage 1: Review and Categorization of Existing Recommendations

Published recommendations on the prevention and management of PIs were identified through an electronic search and consultation with experts between March and July 2017. Keywords for the search were combined from three different domains. The first was related to PIs (ie, pressure ulcers, pressure injuries, decubitus, pressure sores, bedsores, and skin problems), the second was related to self-management (ie, prevention, detection, treatment, self-management, reduction, and risk factors), and the third was related to SCI (ie, spinal cord injury, tetraplegia, quadriplegia, and paraplegia). The online search applied these keywords in both English and German languages. The search was performed in Google as well as PubMed. The research team extracted all recommendations for the prevention and management of PIs that were directed toward or could be applied by community-dwelling individuals with SCI.

The review obtained a comprehensive collection of recommendations that were screened and synthesized (ie, similar recommendations from different sources were merged). The results of stage 1 were presented in a document that was sent to all participants of the consensus meeting for preparation.

### Stage 2: Consensus Meeting

A purposive sample of health professionals and community-dwelling individuals with SCI were invited to participate in a consensus meeting [53]. With the help of SCI-specialized medical doctors, we identified health professionals who may have experience with PIs in individuals with SCI. For the recruitment of those working in the inpatient setting, we contacted the different departments of the four SCI rehabilitation centers in Switzerland and requested for collaboration. For the recruitment of health professionals working in the outpatient setting and individuals with SCI, we relied on informal networks. Through this process we contacted a total of 35 individuals, and they were offered two possible dates for the consensus meeting.

The final sample of 15 participants [53] included SCI-specialized medical doctors, nurses, wound experts, psychologists, occupational therapists, physiotherapists, and nutritionists who were from different parts of Switzerland and working in SCI rehabilitation centers in Switzerland, as well as home care providers, home care counsellors, representatives from an accident insurance fund, and individuals with SCI. Table 1 presents the participants’ characteristics.

The consensus meeting was grounded in a systematic consensus planning process that helps to prioritize issues of a different kind during experts’ discussions [55]. The meeting lasted one day and was structured in two parts. A person experienced in consensus meetings moderated the plenary sessions. Three persons facilitated the working groups. They were trained for the technical tasks (eg, dealing with the voting system) and were knowledgeable about the project.

**Table 1:** Characteristics of participants in the consensus meeting.

Role	Workplace (for HPs^a^)	Working experience as a HP (years)/Years as a wheelchair user	Year of birth	Gender
SCI^b^-specialized medical doctor	Swiss Paraplegic Centre	15	1962	M
SCI-specialized medical doctor	Clinique de réadaptation romande	14	1972	M
SCI-specialized nurse/wound expert	Swiss Paraplegic Centre	18	1966	F
SCI-specialized nurse/wound expert	Clinique de réadaptation romande	16	1974	M
SCI-specialized nurse/wound expert	Clinique de réadaptation romande	2	1992	F
SCI-specialized nurse/wound expert	SCI-specialized counseling service	14	1973	F
Occupational therapist	REHAB Basel	16	1971	F
Physiotherapist	Swiss Paraplegic Centre	18	1965	F
Nutritionist	Swiss Paraplegic Centre	2	1991	F
Psychologist	Swiss Paraplegic Centre	7	1964	F
Psychologist and person with SCI	Balgrist Klinik	28/33	1957	M
Home care provider	Home care service	8	1990	F
Home care counsellor	SCI-specialized counseling service	3	1972	F
Representative from an accident insurance fund	Swiss Accident Insurance Fund (Suva)	8	1967	M
Person with SCI	N/A^c^	N/A/27	1970	M

^a^HP: health professional.

^b^SCI: spinal cord injury.

^c^N/A: not applicable.

#### Consensus Meeting Part I: Recommendations Selection

The participants were divided into two working groups (whenever possible, a representative for every profession and a person with SCI were included in each working group). Moreover, professionals who worked together were included in different groups. They were asked to discuss one by one the recommendations derived from stage 1 and to vote by show of hands in favor of or against their inclusion in the set of recommendations to be implemented in the app. The vote should be based on the relevance and applicability of the recommendations for community-dwelling individuals with SCI. The facilitator of each group was in charge of taking notes on the discussions and carrying out the vote with the help of an ad-hoc technological infrastructure. A Microsoft Access (2010, version 14.0; Microsoft Corp, Redmond, Washington, USA) database containing the list of recommendations resulting from stage 1 was developed prior to the consensus meeting. Every participant voted in favor or against inclusion of each of the recommendations. The facilitator entered the sum of individual votes into the Access database. Based on this sum, a percentage of agreement for including each recommendation was computed. After this first vote (vote A), it was possible to merge the votes of the working groups and retrieve from the system the list of recommendations divided into recommendations to be included, recommendations to be excluded, and recommendations that were ambiguous*.* As the consensus method is based on a democratic debate and judicial model [53], the recommendations voted on below 40% were excluded, the recommendations voted on above 75% were included, and the recommendations voted on between 40% and 75% were considered ambiguous. These thresholds have been defined based on the experience of previous consensus meetings. The last group of recommendations was discussed in a plenary session in which all participants could argue in favor of or against their choice. After this exchange, the working groups met again to vote on the ambiguous recommendations (vote B). The recommendations were included, excluded, or considered ambiguous following the same rules as in vote A.

Moreover, during the group discussions, the participants had the opportunity to indicate that a recommendation needed specification. This was mostly the case when the recommendation was deemed to be too generic or when its applicability for community-dwelling individuals with SCI was considered unclear or vague. The recommendations that needed specification were collected in a list and further elaborated on in an afternoon session (part II).

#### Consensus Meeting Part II: Recommendations Specification

The participants were divided into three working groups that were stratified by profession, workplace, and affiliation with the previous working groups. As for the morning working groups, whenever possible, we distributed the participants so that at least one representative of every profession and of people with SCI was present in each group. Moreover, professionals who worked together were included in different groups. We also differently mixed the participants with respect to the morning working groups.

Participants further specified the recommendations that were indicated during the previous session as being too vague or unclear to be implemented by community-dwelling individuals with SCI in their daily lives. Each of the three working groups received a list of 20 or 21 recommendations to specify (total 62) and was assigned a sheet of paper presenting a research-based user persona. User personas (Multimedia Appendix 1), which are fictional characters with concrete characteristics and behaviors that are intended to represent different user types, have been used in the user-centered design process for designing software [56,57]. They helped make the specification process concrete, as each group could refer to a vivid portrait.

### Stage 3: Consolidation of Results

After the consensus meeting, the research team together with two experts from the project scientific advisory board consolidated the results by refining them and taking into consideration the input of the participants. For instance, special attention was devoted to the recommendations that remained ambiguous after stage 2. They were screened and sorted out by the research team based on eight logical rules for their inclusion or exclusion. The rules (Textbox 1) referred to the size of the discrepancy between the results of vote A and vote B, and between the two working groups. Additionally, new recommendations were developed for domains that, according to the participants, were insufficiently covered by the existing recommendations. The consolidation stage resulted in a newer and more complete set of recommendations. These recommendations were shared with all the participants of the consensus meeting via email. Feedback from the participants was collected and integrated.

**Logical rules for the inclusion or exclusion of ambiguous recommendations.**

1. If the average of group 1 (G1) and group 2 (G2) in vote A is <40% and in vote B is >40% but <75%, exclude the recommendation.
2. If the average of G1 and G2 in vote A is <40% and in vote B is >75%, include the recommendation.
3. If the average of G1 and G2 in vote A is >75%, average of G1 and G2 in vote B is >40% but <75%, and decrement of vote A-vote B is ≥25%, exclude the recommendation.
4. If the average of G1 and G2 in vote A is >75%, average of G1 and G2 in vote B is >40% but <75%, and decrement of vote A-vote B is <25%, include the recommendation.
5. If the average of G1 and G2 in vote A is >75% and in vote B is >75%, include the recommendation.
6. If the average of G1 and G2 in vote A is >40% but <75% and in vote B is >75%, include the recommendation.
7. If the average of G1 and G2 in vote A is >40% but <75%, average of G1 and G2 in vote B is >40% but <75%, and increment of vote A-vote B is ≥25%, include the recommendation.
8. If the average of G1 and G2 in vote A is >40% but <75%, average of G1 and G2 in vote B is >40% but <75%, and increment of vote A-vote B is <25%, exclude the recommendation.

## Results

### Stage 1: Review and Categorization of Existing Recommendations

The sources presented in Table 2 have been identified, and their documents have been systematically reviewed [58-65]. The recommendations extracted from the documents were categorized by applying a deductive-inductive approach. At first, the recommendations were ordered according to the four categories defined by Keast et al (ie, appropriate support surfaces, regular repositioning of the patient, optimizing nutrition, and skin care) [66]. This categorization, however, was not exhaustive. We therefore started an inductive process by grouping together those recommendations that were not covered by the categories defined by Keast et al. We created new categories until all recommendations belonged to one category. We then revised the categories with the aim of reducing their number. We compared among each other the recommendations included in every category and with those included in other categories, and when possible, we merged the categories. Following this procedure, we reached the number of 12 categories. This procedure is similar to the basic rule of the constant comparative method often used in qualitative research, namely the comparison of a new incident with the previous incidents coded in the same category [67].

The result of the review and recommendation categorization was a list of 130 recommendations for the prevention and management of PIs by individuals with SCI organized in relation to the following topics: (1) Support surface (code A); (2) Repositioning (code B); (3) Nutrition (code C); (4) Skin care (code D); (5) Skin assessment (code E); (6) Exercising (code F); (7) Collaboration with health professionals or caregivers (code G); (8) Transfers (code H); (9) Clothing (code I); (10) Body function and structure (code J); (11) Personal factors (code K); and (12) General (code L). The orders of the categories and recommendations within a category do not reflect a priority order. The recommendations were then collected in a preparatory document, which was sent to the participants prior to the consensus meeting.

**Table 2:** Documents reviewed for the identification of recommendations.

Source	Document
Deutschsprachige Medizinische Gesellschaft für Paraplegie (DMGP) (http://www.dmgp.de/)	Querschnittspezifische Dekubitusbehandlung und-prävention (2017) [58] Psychologische Aspekte in der Dekubitusprophylaxe (2012) [59]
Ontario Neurotrauma Foundation (ONF) (http://www.onf.org)	Canadian Best Practice Guidelines for the Prevention and Management of Pressure Ulcers in People with Spinal Cord Injury. A Resource Handbook for Clinicians (2013) [60] Preventing and Treating Pressure Sores. A Guide for People with Spinal Cord Injury (2015) [61]
European Pressure Ulcer Advisory Panel (http://www.ePIap.org/); National Pressure Ulcer Advisory Panel (http://www.nPIap.org/); and Pan Pacific Pressure Injury Alliance (EPIAP-NPIAP-PPPIA) (http://www.internationalguideline.com/)	Prevention and Treatment of Pressure Ulcers: Quick Reference Guide (2014) [62]
Spinal Cord Injury Research Evidence (SCIRE) (www.scireproject.com)	Pressure Ulcers Following Spinal Cord Injury (2014) [63]
International Spinal Cord Society (ISCoS) (http://www.iscos.org.uk/)	Textbook on Comprehensive Management of Spinal Cord Injuries, chapter 48 (2015) [64]
Schweizer Paraplegiker-Zentrum (SPZ) (http://www.paraplegie.ch)	Patientenaufklärung, Druckstellen-Dekubitus - V1.0 [65]

### Stage 2: Consensus Meeting

#### Consensus Meeting Part I: Recommendations Selection

**Figure 1:**
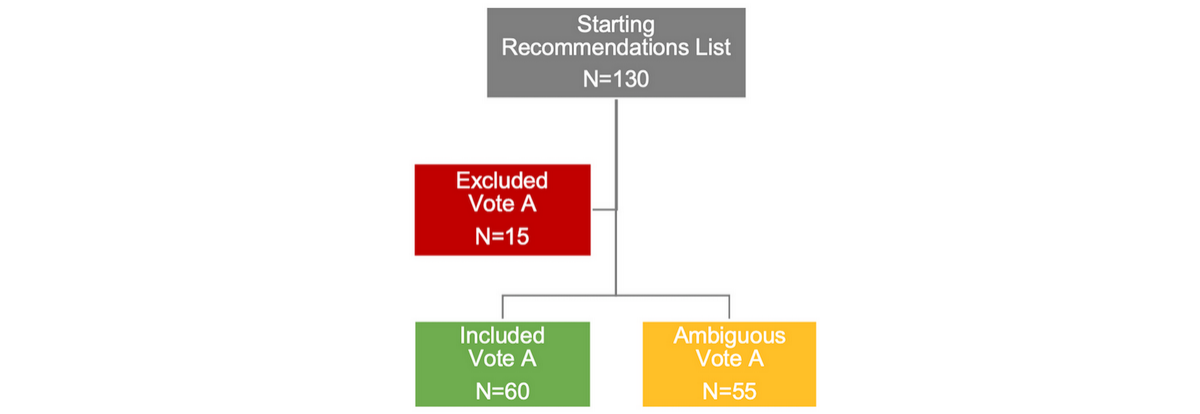
Results from vote A.

Figure 1 shows the results of vote A. From the original list of 130 recommendations, 15 were excluded and 60 were included in the final set of recommendations for implementation in the app. The remaining 55 recommendations, with votes ranging between 40% and 74%, fell into the category of “ambiguous” and were subject to a second vote (vote B).

**Figure 2:**
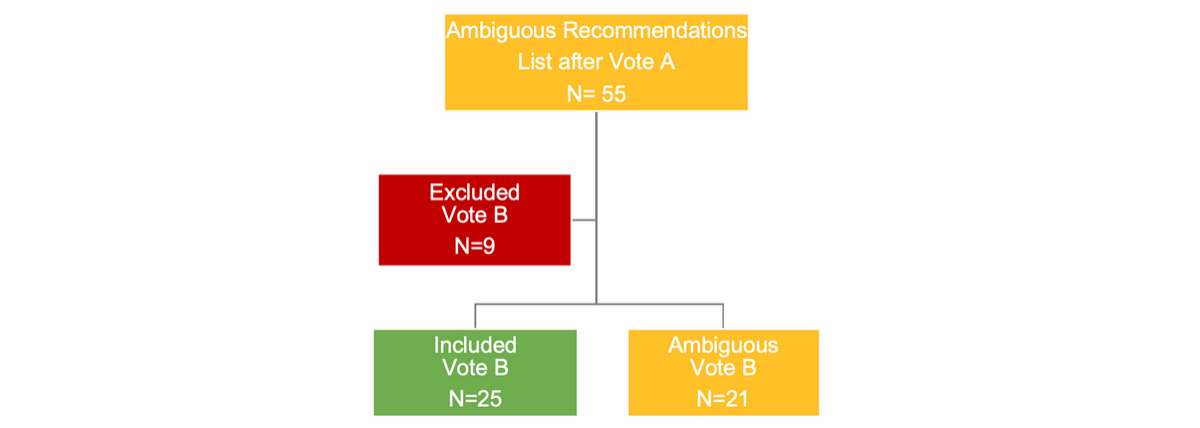
Results from vote B.

Figure 2 shows the results of vote B. Of 55 recommendations, nine were excluded and 25 were included in the final set of recommendations for implementation in the app. Twenty-one recommendations again fell into the category of “ambiguous.” These recommendations were no longer discussed by the participants during the consensus meeting, but were later examined by the research team.

#### Consensus Meeting Part II: Recommendations Specification

A total of 62 recommendations needed specification. The list was composed of recommendations indicated by the working groups as well as recommendations indicated *a priori* by the research team. During the specification phase, different solutions for further clarification of the recommendations were defined by the groups. Most of the recommendations were specified by adding a further explanation of the action to take or by referring to additional criteria for the correct implementation of the recommendation. Approximately one-quarter of the specifications referred to the need to combine complementary recommendations. Experts suggested that a few of the recommendations should be specified by having a dedicated information section about the topic in the app. The specifications were gathered by the research team and further used in the development of evidence-based content for the app.

### Stage 3: Consolidation of Results

#### Decision on Ambiguous Recommendations

The research team examined the 21 recommendations that remained ambiguous after vote B. The ex-post examination resulted in the inclusion of seven recommendations in the final set and the exclusion of 14.

#### Expert Consultations on the Category of Nutrition

The review of existing recommendations (stage 1) resulted in three recommendations for the category of nutrition. These recommendations were debated considerably in the working groups during the consensus meeting (stage 2), as they were considered unsatisfactory. Although participants recognized the importance of nutrition as a risk factor for PIs, they criticized the incompleteness of the presented recommendations and their inability to depict the complexity of nutrition advice in relation to the prevention and management of PIs in individuals with SCI. Hence, the participants agreed with the research team to set up a working group composed of nutritionists and SCI-specialized medical doctors to develop new comprehensive nutrition recommendations. Table 3 provides details of the characteristics of the health professionals who were consulted to develop the recommendations about nutrition.

**Table 3:** Characteristics of the participants consulted for recommendations on nutrition.

Role	Working place	Years of working experience	Year of birth	Gender
Nutritionist^a^	Swiss Paraplegic Centre (Nottwil)	2	1991	F
Nutritionist	Swiss Paraplegic Centre (Nottwil)	6	1980	F
Nutritionist	Swiss Paraplegic Centre (Nottwil)	13	1976	F
SCI^b^-specialized medical doctor	Swiss Paraplegic Centre (Nottwil)	21	1966	F

^a^Took part also in the consensus meeting.

^b^SCI: spinal cord injury.

Multiple sessions of expert consultation were conducted with the aim of developing nutrition recommendations that account for the complex interaction between SCI management and PI prevention. These consultations took place between the end of 2017 and the middle of 2018. Based on previously examined and new sources [62,68-74], the nutritionists developed a new set of recommendations that encompassed information on drinking, weight, and nutrition. The proposed recommendations were then discussed and finalized in collaboration with SCI-specialized medical doctors. In total, six new nutrition recommendations were developed. They were circulated among the participants in the consensus meeting before being added to the final set of recommendations.

The final set of recommendations is presented in Multimedia Appendix 2. It includes 98 recommendations that synthesize evidence-based recommendations for the prevention and management of PIs for community-dwelling individuals with SCI.

A checklist for the process of participatory selection of the evidence to ground a self-management app is presented in Textbox 2.

**Checklist of the process of participatory selection of the evidence to ground a self-management app.**

**Step 1: Systematic review and categorization of existing evidence**

- Theoretical
  Broadly consider the concept of self-management for the respective health condition
  Adopt a holistic perspective for care (bio-psycho-social)
- Methodological
  Consult experts to identify the relevant sources for clinical guidelines
  Identify and screen grey, scientific, and practice-oriented literature
  Expand the search to different languages if possible
  Synthetize the evidence (ie, merge and categorize it). The use of existing classifications might be of help.
- Practical
  Draft a concise document summarizing the available evidence to be delivered to the participants of step 2 for preparation

**Step 2: Consensus meeting**

- Theoretical
  Use an interdisciplinary approach
  Adopt a holistic perspective for care (bio-psycho-social)
  Review models of consensus building and strategies of conflict resolution
- Methodological
  Identify relevant experts for participation in the consensus meeting
  Balance the mix of experts in the consensus meeting (eg, in terms of position, years of working experience, age, and gender)
  Provide the participants with user personas (fictional characters with concrete characteristics and behaviors that are intended to represent different user types)
  Systematize the process of evidence selection to reach democratic decisions
  Be attentive to potential gaps in current evidence pointed out by participants
- Practical
  Facilitate participants’ preparation for the meeting by delivering a concise document synthetizing the evidence and explaining the process
  Train the moderator and facilitators of the working groups in advance (eg, to ensure that all participants express their opinion)
  Provide support with a technological infrastructure to facilitate the voting and the calculation of the vote results
  Allocate enough time for the different tasks and plenary discussions

**Step 3: Consolidation of results**

- Theoretical
  Use an interdisciplinary approach
  Adopt a holistic perspective to care (bio-psycho-social)
- Methodological
  Compare and contrast the results with existing recommendations to identify potential gaps or “blind spots”
  If needed, organize ad-hoc expert consultations
- Practical
  Prepare a report explaining how results were reached
  Elicit participant validation

## Discussion

### Principal Findings

This article proposes a procedure for the participatory identification of evidence-based content to ground the development of a self-management app. To our knowledge, this is one of the first attempts to apply a structured procedure for the participatory identification of evidence-based content for a self-management app in the field of SCI. The procedure consists of the following three steps: review of the literature, consensus meeting, and consolidation of the results (including, for instance, a set of expert consultations, if needed).

Our methodological approach raises two challenges that can hinder the development of evidence-based mHealth interventions. First, it has to be noted that sometimes the literature itself presents contradictory evidence [52,75,76], as the field of medicine is in continuous evolution. This underscores the challenge for clinicians and app developers in terms of identifying evidence-based knowledge on a topic. Thus, the involvement of experienced health care professionals might be a valuable means to assess the available evidence, contextualize evidence and recommendations, identify gaps, and suggest pragmatic solutions [77-79].

The second challenge is to select relevant and applicable evidence for people living in the community. In particular, this study stresses the challenge of selecting the evidence base for the prevention of a complication in the context of a complex chronic condition. Indeed, when selecting the prevention measures for PIs, experts have to take into consideration all aspects of self-management as well as feasibility issues. For instance, in the case of the prevention of PIs in individuals with SCI, it was mentioned during the working group that hydration is very important for preventing PIs; however, liquid intake often requires catheterization, which, in turn, can increase the risk of bladder infections. Similarly, doing pushup exercise to relieve the skin is good for preventing PIs, but it could cause damage to the shoulders in the long term. These examples illustrate the complexity and sometimes conflicting nature of evidence-based recommendations that are feasible for community-dwelling individuals and that ensure a comprehensive approach to the self-management of SCI. Indeed, systematic reviews and meta-analysis offer valuable synthesis of the evidence [80], but they often have a narrow focus (eg, one complication), and in many cases, they only report on experimental studies, which, owing to their rigor, avoid biases (eg, confounding factors and selection bias), but do not consider real-life situations [81]. In order to overcome these limitations and achieve a comprehensive approach to self-management, it is fundamental for experts from all relevant specialties as well as the persons affected by the health condition to be involved in the selection of the evidence for mHealth interventions. The combination of interdisciplinarity and lived experiences ensures that all perspectives are represented in the discussion. However, for the discussion to be constructive and achieve agreement on a shared decision, a structured process is needed. A consensus meeting represents a valid method to synthesize information and enable decisions to be made when published information is inconsistent or inadequate [82], and it is widely used in medical and health services research [83-85].

### Limitations

We have to acknowledge a few limitations of our study. The first one is related to the selection of recommendations, as we searched only for recommendations in English and German. We also focused on recommendations specific to SCI and PIs, not considering, for instance, other recommendations on SCI in general or on PIs in other populations. The second limitation is linked to the participants in the consensus meeting. All relevant stakeholders were represented; however, the participant mix could have been more balanced (eg, there were many nurses and only one occupational therapist). In addition, the consensus meeting was held on only one day. This resulted in focused discussions on many relevant aspects of the recommendations, but it was very intense for all participants. Having more time at our disposal could have also allowed an additional discussion and voting round to avoid concluding the meeting while still having some ambiguous recommendations, which the research team later needed to clarify. We also acknowledge that the procedure used has not been compared with another procedure and has not been evaluated. However, the commitment of the participants during the procedure showed that the participatory approach was positively received.

### Strengths

Although this study had the above-mentioned limitations, it is important to acknowledge some of its strengths. The methodological choice of holding a consensus meeting has been proven to be highly valuable, as its structured process guarantees a democratic discussion and a judicial model [53]; hence, it provides a viable and transparent option for a true participatory design process. Three other strengths that we want to highlight helped the procedures of stage 2 to run more smoothly. First, the selection of experts through other professionals and an informal network proved to be highly valuable, and it provided credibility to our invitation. Moreover, being aware of time constraints, we condensed the consensus meeting activities in one day and provided stakeholders with two dates as options. Second, it should be noted that for constructive discussions during the consensus meeting, participant preparation for the session was extremely important (ie, having read the preparatory document describing the procedure and the list of recommendations resulting from stage 1). Third, having an efficient and automated voting system was essential for ensuring that the results of one vote were quickly available for the next round (plenary or group discussion). This case study proved the value of the presented procedure; however, as this was a demonstration study, there is a requirement for further studies to validate the approach.

### Conclusion

Considering that people need evidence-based information to make informed decisions and participate in health [29], this study may be valuable for improving the quality of mHealth interventions as it detailed the participatory procedure needed for the selection of the scientific evidence that forms the basis of mHealth content. In particular, this procedure might be useful in the selection of evidence-based content in the case of complex chronic health conditions, for which every recommendation needs to be evaluated and considered in light of all other self-management requirements. Hence, agreement among all experts and affected individuals on which evidence is to be included is essential.

## Appendix

Multimedia Appendix 1 User personas.

Multimedia Appendix 2 Final set of recommendations for pressure injury prevention and management.

## Abbreviations

eHealth: electronic health
mHealth: mobile health
PI: pressure injury
SCI: spinal cord injury
