# Supplementary material for: Selecting Evidence-Based Content for Inclusion in Self-Management Apps for Pressure Injuries in Individuals With Spinal Cord Injury: Participatory Design Study
Source: JMIR Mhealth Uhealth. 2020 May 20;8(5):e15818. doi: 10.2196/15818 (PMC7270844; doi:10.2196/15818)
Supplement: Multimedia Appendix 1 [file mhealth_v8i5e15818_app1.pdf]

USER PERSONA FOR THE CONSENSUS MEETING/APP DEVELOPMENT

REGULA

Regula, 35 years old, working as accountant part time 30%, married and mother of a 3–year-old girl, paraplegic since 15 years due to a horse-riding accident

Typical day: Regula lives in Lucerne with her family. She usually wakes up at 7 am to have breakfast with her husband before waking their daughter. At 8:30 am she leaves the apartment to bring her daughter to the kindergarten. She then goes back home to work. She lives next to her parents, who regularly pick up the granddaughter at noon to have lunch all together. In the afternoon Regula takes care of her child. They go to the playground and meet with other kids and mothers of the neighborhood, they go grocery shopping, play at home or visit friends. In the evenings, Regula likes to cook dinner for the family. Once a week, she takes an afternoon free and while her husband takes care of their daughter, she spends some time with a friend and has a massage. On this day, they usually order pizza or Thai food for dinner. During the weekend they like to go (hand)biking at the lake.

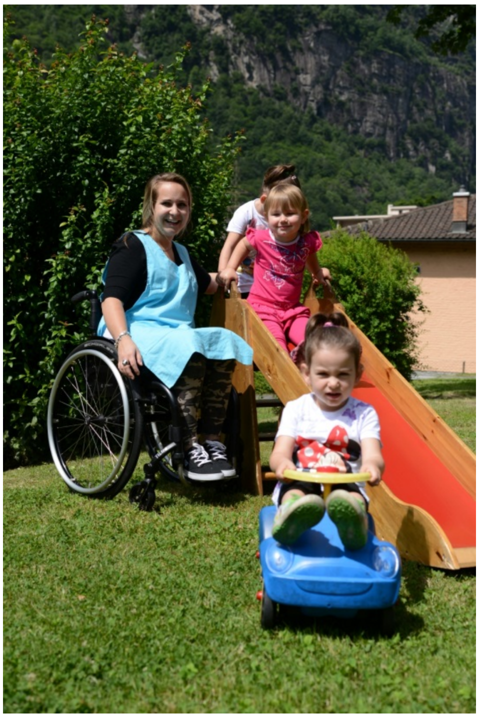

Challenge: now that her daughter does not rest in the afternoon anymore, Regula no longer has the chance to rest either. Currently she spends more than ten hours a day in her wheelchair.

Objective: she used to lay down during the afternoon to relieve the skin from pressure. Now she wonders how to compensate the lack of bedrest.

Relationship with technology: advanced user

Must does: remind her to take care of herself and find alternative ways to rest

Must doesn’t: make her too obsessed with the device as she wants to avoid her child to use the phone

USER PERSONA FOR THE CONSENSUS MEETING/APP DEVELOPMENT

HANS

Hans, 73 years old, retired, living alone, paraplegic since 37 years due to a job-accident on a construction site

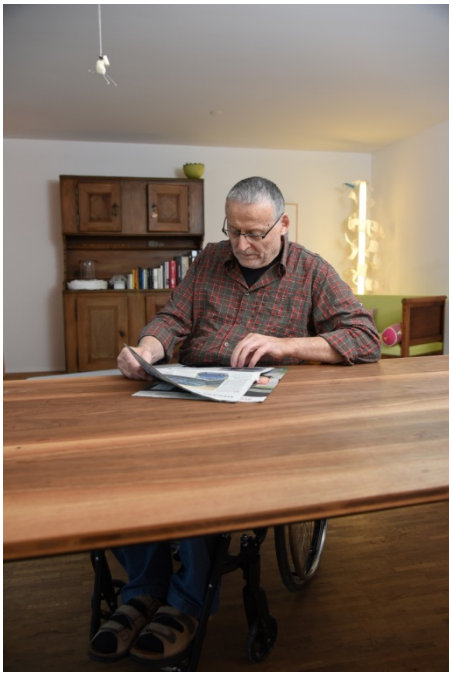

Typical day: Hans lives alone in a village in the canton Basel. He has a dog and he wakes up to walk it every morning. He is a former construction worker, but since the accident he did not get back to his job. He converted his passion for construction into the passion for scale modeling and built number of small helicopters and airplanes. This hobby keeps him busy several hours per week. However, recently he is frustrated because he started to have some problems with fine motor function and he noticed that he works slower and with less precision than before. The son used to come every evening for dinner, but since he got married and moved to St. Gallen he visits only once per week. Hans suffers from seeing his son so little. He enjoyed planning the menus, going grocery shopping and cooking for the two of them. Cooking only for himself seems to him a waste of time. He prefers to have some bread and cheese in front of the TV.

Challenge: because of ageing he is experiencing a lack of strengths that impacts his ability to transfer. A couple of days ago, while transferring from the wheelchair to the car he bumped into the car seat.

Objective: Hans developed a pressure ulcer. He needs now to monitor it. Since in his wheelchair he sits on the pressure ulcer, he has to lay down more often to relieve the skin during the day, to avoid that it gets worse. He wonders how to best monitor his pressure ulcer.

Relationship with technology: basic user, new to smartphone

Must does: Interface should be clear to use and navigation should be self-explanatory, intuitive. The technology should stimulate Hans in taking care of himself (“Have you had a proper meal today?”).

Must doesn’t: should not have too much to read on a screen because the smartphone is not very large and reading is getting difficult with ageing

USER PERSONA FOR THE CONSENSUS MEETING/APP DEVELOPMENT

MARTIN

Martin, 49 years old, male, part-time high school science teacher, living with his partner, paraplegic since 20 years because of a ski-accident

Typical day: Martin recently moved with his partner in a new apartment in the region of Lausanne. Two days per week he works at school, where he teaches natural sciences (six hours a week). Thanks to the advanced and well-organized web portal of the school, he can carry out most of the administrative and preparation work at home. For instance, he takes part in the staff meetings in remote via Skype. Martin is really passionate about information and communication technologies, and three times a week he collaborates with a cooperative society for the development of open source technologies which can enable SCI individuals to accomplish different tasks through the implementation of integrated voice control. Since his partner is also a teacher, they can profit from many holiday weeks during the year. They share the passion for travelling and planned to visit all European capitals in the next years. One of his dreams would be to rent a car and do a coast to coast trip in the US, from NYC to LA. With his partner he is checking if such a trip is feasible also for a person with SCI.

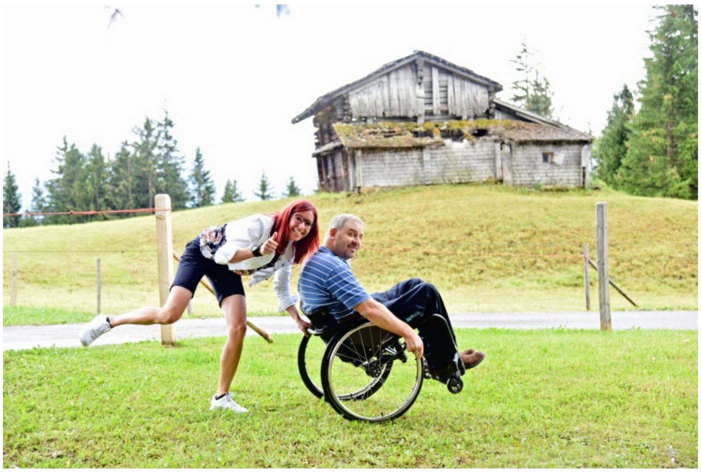

Challenge: In the last year he has developed several superficial pressure ulcers, most of the time while travelling. Two weeks ago he developed again a superficial pressure ulcer during a weekend abroad and once at home he found out that his wheelchair cushion was worn out. He is currently trying a new one.

Objective: He currently has to perform visual and tactile skin inspection more frequently as he is trying new material. In addition, in the future he will need to remember to check his cushion regularly to identify signs of deterioration. Considering his passion for travelling and his plans and dreams, he also has to find out how to prevent pressure ulcers when travelling.

Relationship with technology: proficient user, also skillful in programming

Must does: the technology should enable personalization/adaptation to user needs

Must doesn't: the app should be able to communicate and be compatible with other systems/devices. Also, it should not be too heavy or consume too much battery as he wants to be able to use it efficiently also while travelling.

USER PERSONA FOR THE CONSENSUS MEETING/APP DEVELOPMENT

JOHN

John, 18 years old, living with her parents and two siblings, tetraplegic since one year after a car accident

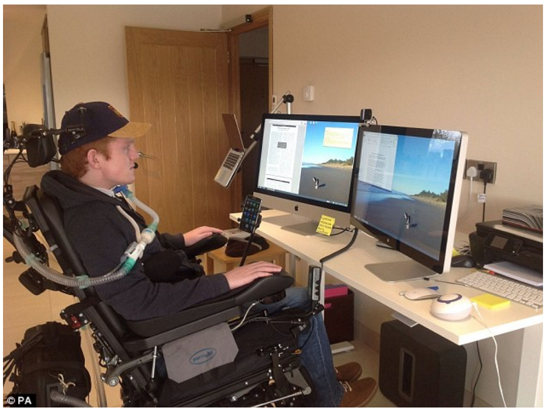

Typical day: John lives in the family house in a village in the Canton Fribourg. He quit high school after the accident and has to find out what he would like to do. Currently he spends his days at home, watching series on Netflix, playing online videogames and smoking on the balcony. Keeping contacts with his former friends is not easy, as he used to meet up with them for soccer training and for drinks on the weekends. He is still in contact with them, but prefers the WhattsApp group chat to meeting with them because he is afraid of having to experience their compassion or embarrass. He misses the adrenaline of the competition and the feeling of being part of a team. During rehabilitation he watched the rugby championships in Nottwil and found it cool, but now that he is at home he has the feeling that doing sport would require too much of an effort. His parents and siblings try to involve him in family activities, but he would just like to go back to his previous life, much easier and spontaneous than the current one. He would like to get the driving license to become more independent. John sees a physiotherapist twice a week with the aim of maintaining joint mobility and improving her upper limb and hand function, which is very important for his independence in daily tasks.

Challenge: not having a clear routine during the day, he regularly forgets to ensure pressure reliefs and repositioning and is upset when his mom reminds him

Objective: he needs to be reminded of doing weight shifts to ensure pressure redistribution; he would need to be stimulated for practicing some sport

Relationship with technology: basic user (familiar only with communication technology like social media)

Must does: In collaboration with his healthcare team, the technology should help his develop an individualized prevention plan (i.e. select a technique and frequency that best meets his needs). In addition, the technology should be usable even with his limited hand function.

Must doesn't: be obsessive and have only annoying reminders as he still needs to accept his condition and rather become more empowered

USER PERSONA FOR THE CONSENSUS MEETING/APP DEVELOPMENT

ANNA

Anna, 18 years old, student at the high school, living with her parents and two siblings, tetraplegic since one year after a horse-riding accident

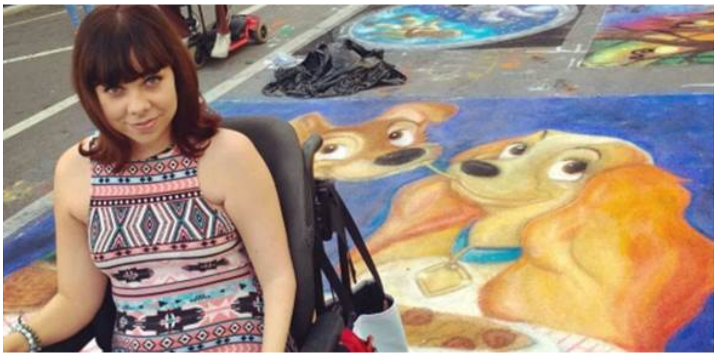

Typical day: Anna lives in the family house in a village in the Canton Fribourg. She wakes up at 6 am to get ready for school. With the support of her mum, she gets up, wash herself and dresses before having breakfast with her siblings. Her dad drives her to school every morning. She attends the 11th grade at the local high school. Her best friend never left her alone after her accident, and she comes to visit Anna almost every day: they do their homework and prepare for tests together, spend time chatting, listening to the last hits and watching YouTube videos. Anna sees her physiotherapist twice a week after school with the aim of improving her upper limb and hand function, which is very important for her independence at home as well as at school. She loves movies since she was a child, her favorites are fantasy movies. On Thursdays is the Family Movie Night and each member of the family, in turn, suggests a movie to watch. She spends most of the weekends with her family and, although they try to involve her in as many activities as possible, she misses horses and the time spent at the stables. She is currently looking for information about possibilities for horse therapy.

Challenge: back at home and school, she needs to learn how to integrate pressure ulcer prevention in her daily life.

Objective: Since Anna does not go back home for lunch, she needs to plan a rest during school time. In addition, she needs to be reminded of doing weight shifts to ensure pressure redistribution.

Relationship with technology: basic user (familiar only with communication technology like social media)

Must does: The app should become a companion that lies "in the back" as she does not need it yet, but she could maybe in the future and due to a change in her life. Therefore the app should offer something appealing in terms of knowledge, connection or other functions that may be appreciated by her

Must doesn't: remain "too silent" for long thus to invite her to forget about it and even to delete it
