# Supplementary material for: Selecting Evidence-Based Content for Inclusion in Self-Management Apps for Pressure Injuries in Individuals With Spinal Cord Injury: Participatory Design Study
Source: JMIR Mhealth Uhealth. 2020 May 20;8(5):e15818. doi: 10.2196/15818 (PMC7270844; doi:10.2196/15818)
Supplement: Multimedia Appendix 2 [file mhealth_v8i5e15818_app2.docx]

**Multimedia Appendix 2.** Final set of recommendations for pressure injury prevention and management.

| **Code^a^** | **Recommendations** |
| --- | --- |
| A-1 | Mattresses must be selected individually after prior testing^b^. |
| A-2 | Assistive devices, such as seat cushions and mattresses, do not replace, under any circumstances, necessary relief, repositioning, and skin checks!^b^ |
| A-3 | Individualize the periodic re-evaluation of a wheelchair surface and other equipment for posture and pressure redistribution^b,c,d,e,f^. |
| A-4 | Replace seating equipment and support surfaces according to manufacturer’s recommendations or sooner if equipment demonstrates any signs of deterioration, including but not limited to wear, cracking, and allowing bottoming out^c^. |
| A-5 | Schedule reassessment at least every 2 years or sooner if any of the following occur: health status changes, including weight and medical changes; changes in the functional status; equipment wear or disrepair; pressure ulcer development; and changes in the living situation^c^. |
| A-8 | Select a pressure redistribution seating cushion that provides contour, uniform pressure distribution, and high immersion^d^; promotes adequate posture and stability in resting and dynamic positions^c,d^; permits air exchange to minimize temperature and moisture^c,d^; is comfortable^c^; and has a stretchable cover that fits loosely on the top cushion surface and conforms with body contours^d,f^. |
| A-9 | Implement a trial of at least 24 hours and preferably several days to ensure the equipment both addresses pressure and microclimate issues and meets functional and lifestyle needs^c^. |
| A-10 | Cushion maintenance: learn how to care for and maintain the wheelchair cushion; monitor the cushion for signs of wear at an appropriate frequency; set up the cushion properly, including orientation and monitoring for bottoming out; and replace the cushion if it is deteriorating^c^. |
| A-11 | Do not sit on a hard chair or lay down on a wooden floor for a long time^c^. |
| A-15 | Do not use synthetic sheepskin pads^d^; cut-out, ring, or donut-type devices, such as cushions and mattresses, because the edges of these devices create areas of high pressure that may damage tissue^c,d^; intravenous fluid bags^d^; and water-filled gloves^d^. |
| A-16 | Minimal interference between the support surface and the person using it maximizes the effectiveness of a support surface in redistributing pressure. Additional layers, such as incontinence pads, lambskin, blankets, and pillows, can all reduce the immersion or envelopment capabilities of the support surface, reducing the pressure-redistributing properties of the support surface. The thicker the interference, the greater the reduction in effectiveness of the support surface^c^. |
| A-18 | Consider using a pressure-redistributing surface on the commode or toilet to minimize pressure ulcer risk^c^. |
| A-21 | Consider the need for moisture and temperature control when selecting a support surface and a support surface cover^d^. |
| B-1 | Consider the use of electrical stimulation to decrease ischial pressure and increase blood flow to tissues^c^. |
| B-2 | Use prone positioning to stretch the hips and trunk while offloading the buttock region, including the ischial tuberosity, sacrum, and coccyx^c^. |
| B-3 | Maintain proper positioning and postural control^d^. |
| B-4 | Provide adequate seat tilt to prevent sliding forward in a wheelchair or chair, and adjust footrests and armrests to maintain proper posture and pressure redistribution^d^. |
| B-6 | Avoid sitting in bed. Transfer to a sitting surface that is designed to distribute pressure properly in the seated position^c^. |
| B-7 | Limit head-of-bed elevation to 30° for an individual on bedrest^c,d^. |
| B-9 | When lying down, protect your heels. Lift your heels up with a wedge cushion or let them rest over the edge of the mattress^c^. |
| B-10 | When lying down, use pillows for support on your side and stomach. Take pressure off your knees and hips with cushions or pillows. Make sure you feel comfortable^c^. |
| B-13 | Pressure relief in the bed through various positions (lateral position and prone position)^g^. |
| B-15 | Pressure can be redistributed intentionally when an individual uses the upper body or arms to intentionally shift his or her weight. Such maneuvers include side and forward leaning and whole-body lifts^c,g^. |
| B-16 | Lean forward or to the side, as this produces more complete and prolonged pressure reductions than lifting vertically^c^. |
| B-19 | Tetraplegics or older persons may relieve pressure by leaning forward with the chest towards the thighs or by doing sideways bending while fixing the trunk on the contra lateral side with the upper limb blocked under the handle of the chair. When active pressure relief is not possible, the caregiver will have to assist the patient in obtaining pressure relief^f^. |
| B-21 | Consider power weight-shifting technology when other methods, such as active pressure redistribution and pressure redistribution through functional movements, are not effective or not possible^c^. Use power weight-shifting technologies, such as tilt, recline, and stand, frequently throughout the day to reduce the effects of sitting pressure on the bony prominences of the buttocks^c^. |
| B-24 | Whatever technique is used, weight bearing areas should be routinely pressure relieved for 15-20 s every 20-30 min or for 60-120 s every 60 min to allow recovery of blood flow and oxygenation^f^. |
| B-25 | Ideally, you should redistribute your weight every 15-20 min^c,g^. To return tissue oxygenation to unloaded levels, a pressure-relieving movement must be at least 1-2 min in duration. Sustaining a vertical lift for this length of time is simply not possible for most people with spinal cord injury^c^. |
| B-27 | If redness can no longer be pressed away, it takes time for the skin to recover. If the pressure injury is in a place that is not under pressure during mobilization in the wheelchair, it is possible to continue to sit in the wheelchair during the day, but several breaks are indicated. If the area is under pressure while sitting, bed rest with regular repositioning is necessary^g^. |
| C-1 | Overweight and underweight represent an increased risk for the development of pressure injuries. Determine your ideal weight in co-operation with your doctor or a nutritionist specialized in paraplegia.  The ideal weight for a person with spinal cord injury is more difficult to determine. BMI can be used as a support. Currently, a BMI between 16 and 23 is recommended^d,h^. |
| C-2 | Ideally, check your weight once a month and at least once a year as part of the annual check. Find individualized strategies to control your weight (eg, Landi scales, post office, etc; measure abdominal girth; mark belt hole; "reference trousers;" and wheelchair width)^c^. |
| C-3 | In case of an unwanted weight change of more than 5% (weight gain or loss) within 2-3 months, consult your family doctor. He or she will examine you and possibly refer you to a dietician^g^. |
| C-4 | Make sure you eat a balanced mixed diet based on the recommendations of the Swiss Nutrition Society. Owing to the changed energy and protein requirements associated with a spinal cord injury, the intake of carbohydrates should be slightly reduced compared with the recommendations of the Swiss Nutrition Society and sufficient proteins should be provided^g^. |
| C-5 | There is an increased protein requirement to minimize the risk of pressure injuries. If you do not know your individual protein requirement and how to calculate it and ensure a sufficient intake without greatly increasing your calorie intake, contact a nutritionist specialized in paraplegia.  Example: Individual with paraplegia having a height of 175 cm, weight of 61 kg, and BMI of 20 (0.8-1 g/kg/d protein) requires 49-61 g protein daily^c,d,g^. |
| C-6 | Ideally, you should check your drinking and eating habits once a month. For example, use the website of the Swiss Nutrition Society (http://www.sge-ssn.ch/ich-und-du/tests-zur-ernaehrung/test-zur-lebensmittelpyramide/test-lmp/). Here, you will find a test with 15 questions concerning your general eating behavior. The result shows you at a glance for which food groups you reach, fall short of, or exceed the recommendations.  If the result is inadequate, especially at the level of "dairy products, meat, fish, eggs, and tofu," contact a nutritionist specialized in spinal cord injury^d,g^. |
| D-1 | Affected areas should be kept clean and dry^c,e,f^, especially under medical devices^d^. |
| D-2 | Handle skin gently during cleansing to minimize force and friction^d^. |
| D-3 | Avoid massaging or vigorously rubbing the skin. It may cause inflammation or destruction of tissue^c,d^. |
| D-4 | Cleanse the skin promptly following incontinence or moisture^c,d,e^. |
| D-5 | Special care should be taken around skin creases and natural body folds^f^. |
| D-7 | Use a nonsensitizing, pH-balanced, fragrance-free, alcohol-free emollient moisturizer, as dry skin appears to be an independent and relevant pressure ulcer risk factor^c,d^. |
| E-1 | Conduct comprehensive daily visual and tactile skin inspections during rehabilitation and initial community reintegration, paying special attention to the areas that are most vulnerable to pressure ulcer development^c^. The most common sites for pressure ulcers are the ischia, sacrum, and trochanters. It is also important to assess other bony prominences, including the occiput, heels, ankles, knees, scapula, and elbows^c,e^. |
| E-2 | Use a long-handle mirror for visual inspection^c^. |
| E-3 | Additional skin checks are important after sitting and lying on a new, unknown surface (new mattress and bedding material at home)^e,g^; foreign mattress in a hotel, with friends, in a hospital, etc^e,g^; new wheelchair or seat cushion^c,g^; sports equipment, such as monoski, handbike, racing wheelchair, etc^g^; aircraft seat, transfer seat for aircraft, etc^g^; dentist's chair, examination couch, X-ray table, etc^g^. |
| E-4 | Additional skin checks are important after wearing new clothing (eg, be careful with thick trouser seams) and shoes^g^. |
| E-5 | Additional skin controls are important for skin changes, such as swelling, dry skin/skin cracks, very scaly skin, increased callus, and hardening^g^. |
| E-6 | Inspect the skin in case of accidents (eg, after falling)^g^, worsening of the general health status,^c,d^ or illness (eg, fever, flu, diarrhea, increased spasticity, and infections)^c,g^. |
| E-7 | Additional skin controls are important in hot climates and heavy sweating^g^. |
| E-8 | The most important measure for the prevention of pressure injuries is a regular control-visual inspection and palpation of the skin. Press the reddened area of the skin with your finger. If the area can be pushed away (1-2 s whitish discoloration), further damage to the skin can be prevented by relief. If the area cannot be pushed away (ie, it remains red; if the skin is damaged, it is already a decubitus), a longer consistent relief phase is required. If hardening, scabbing, or blistering occurs, the decubitus is deeper and must be treated urgently, and a doctor’s visit is necessary^g^. |
| E-9 | Tactile inspection should evaluate skin temperature, moisture, and induration, or bogginess^c^. |
| E-10 | Consider taking photographs (or having someone taking them) to monitor pressure ulcer healing over time^c,d^. |
| E-11 | Inspect the skin (under and around medical devices^d^) regularly, at least twice daily^c,d,f,g^, for example, in the morning during personal hygiene and in the evening before going to bed^g^. |
| E-12 | Visually inspect for redness, discoloration, and bruising; changes in texture, such as rashes, dryness, and swelling; and abnormalities, such as cracks, scabs, and blisters^c^. |
| F-3 | Exercise regularly (twice a week for 20 min and stretch)^c^. |
| F-5 | Use active or active-assisted movements to maintain joint range of motion and muscle flexibility, when possible^c^. |
| F-6 | Use conventional passive range-of-motion programs when there is no motor function^c^. |
| F-7 | Participate in regular exercise that is consistent with Canadian physical activity guidelines to optimize muscle strength and endurance, encourage postural alignment, improve cardiovascular endurance, and prevent fatigue and deconditioning^c^. |
| G-1 | Establish pressure relief schedules that prescribe the frequency and duration of weight shifts^d^. |
| G-2 | Get treatment like speaking or medication if depressed or sad^c^. |
| G-3 | When possible, use specialized seating clinics with an interprofessional team of clinicians who have expert training and experience working with people with spinal cord injury to assess and recommend wheelchairs and other sitting support surfaces^c^. |
| G-4 | You and those who help with your care should always watch for signs of pressure injury^c,e^. |
| G-5 | Try to have the same person help you all the time so that they know what is normal for your skin and train her or him^c^. |
| G-7 | The longer a pressure injury remains untreated, the deeper the decubitus and the longer the healing phase. It does not make sense to treat the wound yourself for a long time. Early monitoring and treatment by your family doctor will prevent complications. It is also possible to contact the Schweizer Paraplegiker-Zentrum outpatient clinic^g^. |
| G-8 | Speak to health professionals if you need help with addictive habits^c^. |
| G-10 | Optimize the bowel care routine to minimize time using the commode, and reassess the bowel program if more than 1 hour is required^c^. |
| G-11 | If hardening, scabbing, or blistering occurs and the decubitus is deeper and must be treated urgently, a doctor's visit is necessary (eg, outpatient clinic, ParaHelp)^g^. |
| G-12 | Work with the health care team to develop an individualized prevention plan (ie, select a technique [lifting or leaning] and frequency that best meets your needs)^c,d^. |
| H-1 | Avoid dragging your body; lift your body^c^. |
| H-2 | Keep the path between transfer surfaces clear of obstacles, including armrests, footrests, and clothing guards, to reduce the risk of contact injury^c^. |
| H-4 | Minimize the gap between surfaces. Placing the wheelchair parallel or at a slight angle to the transfer surface creates the smallest gap and transfer distance^c^. |
| H-5 | Choose smooth, low-friction, breathable materials for sheets, pajamas, mechanical lifts, and slings^c^. |
| H-6 | Use care in repositioning your body after transfer to prevent friction and shear from stretching the skin or folding soft tissues during movement^c^. |
| H-7 | Consider adaptive equipment to assist repositioning and transfers, such as sliding boards, bed rails, and trapeze bars. Objects in the immediate environment, such as armrests, backrests, and parts of the wheelchair frame may also be used to assist repositioning and transfers^c^. |
| H-8 | Select and train transfer techniques for all surfaces necessary for daily activities to ensure safe repositioning and minimize skin and tissue damage during movement^c^. |
| H-9 | Remove handling equipment under the body after use^d^. |
| I-1 | Do not wear tight-fitting shoes^c^. Increase the preinjury shoe size by at least a size to compensate for the development of peripheral edema and prevent pressure ulcers on the feet^c^. |
| I-2 | It is also important to check the feet for signs of pressure every 30 min when wearing new shoes^c^. |
| I-3 | Use appropriate clothing without studs, buttons, or thick seams on the buttocks and back^g^. |
| I-4 | If there is spinal cord injury, avoid tight clothing, including underwear^c^. |
| I-5 | Use clothing that are breathable, soft, stretchy, and smooth and have low friction to optimize microclimate control and minimize friction^c^. |
| J-1 | The inability to regulate body temperature due to impaired vasoregulation puts the person with spinal cord injury at risk of hypothermia or hyperthermia, depending on the external temperature. The excessive moisture associated with it can make the skin more prone to bacterial and fungal infection^f^. |
| J-2 | Do not apply heating devices (eg, hot water bottles, heating pads, and built-in bed warmers) directly on skin surfaces or pressure ulcers. Heat increases the metabolic rate, induces sweating, and decreases the tolerance of the tissue to pressure^d^. |
| K-1 | Depression has been linked to various negative outcomes in the spinal cord injury population, including poor adjustment, decreased quality of life, and health problems such as pressure ulcers and urinary tract infections^c^. |
| K-2 | Changes in self-image and self-worth that people experience after spinal cord injury may lead to neglect of the paralyzed parts of the body, social isolation, and depression^f^. |
| K-3 | Be careful with dramatic weightloss^c^. |
| K-4 | Decrease or stop smoking and limit alcohol intake^e^. |
| K-6 | Be aware of additional risk in life events breaking in your daily routines and do not neglect checking your skin regularly nevertheless^c^. |
| K-7 | Be extra aware when there has been a pressure injury during acute rehabilitation or in the previous 3 years^e^. |
| K-10 | Everyone’s skin gets more fragile with age, and muscle strength and movement decrease^d^. |
| L-1 | Electrical stimulation likely helps to prevent pressure injury formation or progression by reducing pressure and increasing tissue oxygenation^c,d,e^. |
| L-2 | Avoid accidentally lying or sitting on objects that could cause pressure injuries (eg, buttons, mobile phones, etc)^g^. Avoid filling pockets with items such as keys and mobile phones^c^. |
| L-3 | Check for existing opportunities to get educated after the acute phase (eg, e-learning)^c,d^. |
| L-4 | Continuous pressure relief requires perseverance and meticulous attention to routine on the part of the patient and the health professionals caring for the patient^f^. |
| L-5 | Develop and implement an individualized continence management plan^d^. |
| L-6 | Find a strategy for achieving optimum pressure relief acceptable to everyone involved^f^. |
| L-8 | Often patients’ responsibility, autonomy, and freedom to live their own life without spinal cord injury-related limitations have to be balanced against the precautions that have to be taken to prevent pressure ulcers^f^. |
| L-11 | Choose pressure-redistribution strategies that are individualized to your needs and lifestyle^c^. |
| L-12 | If redness occurs, immediate relief of the affected area is necessary until the skin has recovered. The more advanced the pressure injury is, the longer the healing process will take^g^. |

^a^The code indicators are as follows: A, Support surface; B, Repositioning; C, Nutrition; D, Skin care; E, Skin assessment; F, Exercising; G, Collaboration with health professionals or caregivers; H, Transfers; I, Clothing; J, Body function and structure; K, Personal factors; L, General.

^b^Deutschsprachige Medizinische Gesellschaft für Paraplegie (DMGP).

^c^Ontario Neurotrauma Foundation (ONF).

^d^European Pressure Ulcer Advisory Panel; National Pressure Ulcer Advisory Panel; and Pan Pacific Pressure Injury Alliance (EPIAP-NPIAP-PPPIA).

^e^Spinal Cord Injury Research Evidence (SCIRE).

^f^International Spinal Cord Society (ISCoS).

^g^Schweizer Paraplegiker-Zentrum (SPZ).

^h^Evidence Anlaysis Library-Academy of Nutrition and Dietetics (EAL).
